# Comparative analysis of viability, proliferation, and mineralization potential of human pulp and osteoblastic cells exposed to different bioceramic endodontic sealers

**DOI:** 10.1016/j.jobcr.2025.01.008

**Published:** 2025-01-14

**Authors:** Marcos Coelho Santiago, Gustavo Henrique de Oliveira Salles, Gustavo Gomes de Lima, Laudimar Alves de Oliveira, Loise Pedrosa Salles

**Affiliations:** a University of Brasilia, Faculty of Health Sciences, Post-Graduation Program in Dentistry, Brasília, DF, Brazil; b University of Planalto Central, Medical School, Brasília, DF, Brazil

**Keywords:** Calcium silicate, Dental materials, Dental pulp, Endodontics, Osteoblasts, Root canal obturation

## Abstract

**Background:**

The present study aimed to compare the viability, proliferation, and mineralization potential of human dental pulp cells (hDPCs) and osteoblasts cell line (Saos-2) after exposure to AH Plus® Bioceramic (AHP-B), Bio-C® Sealer (BIO-C), NeoMTA Plus® (NEOMTA-P), and MTA-FILLAPEX® endodontic sealers (MTA-F).

**Methods:**

All materials were prepared according to the manufacturer's instructions. Before exposing the cells, we measured the release of calcium ions (Ca^2+^) from the dental materials to the culture media once Ca^2+^ can trigger signaling pathways. After that, hDPCs and Saos-2 were exposed to the sealers for MTT assay to assess the cell viability and wound healing to evaluate the cell proliferation. To investigate the potential of mineralization, we assessed the alkaline phosphatase activity and calcium deposition by Alizarin red staining. Statistical analysis was performed using Two-way ANOVA for calcium release and wound healing assays and One-way ANOVA for other assays, with post-test Bonferroni correction. The results were significant when p < 0.05.

**Results:**

The sealers released diverse concentrations of calcium at different times. The hDPCs viability and proliferation were low in the AHP-B group at 24h of exposure (NEOMTA-P ∼ BIO-C ∼ CT > AHP-B > MTA-F), distinct from the osteoblastic cells (NEOMTA-P ∼ AHP-B ∼ CT > BIO-C > MTA-F) and (proliferation: AHP-B > NEOMTA-P ∼ CT > BIO-C > MTA-F). The ALP activity, an early marker of osteogenesis, was higher in hDPCs exposed to NEOMTA-P, while the osteoblastic cells showed higher ALP when exposed to AHP-B.

**Conclusion:**

AHP-B, NEOMTA-P, and BIO-C stimulated osteogenesis in hDPCs and Saos-2 cells, with marked differences between groups. AHP-B showed an improved early stimulation of osteoblastic cells, while hDPCs were more responsive to NEOMTA-P.

## Graphical abstract

**Figure:**
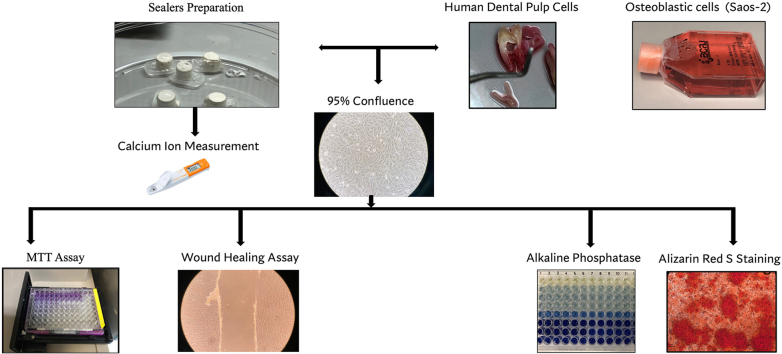

## 1 Introduction

Endodontic sealers play a crucial role in the success of root canal therapy by providing a tight seal between the root canal filling material and the canal walls, preventing bacterial infiltration and promoting the healing of the surrounding tissues.^1^ These materials must ensure biological and mechanical properties, such as biocompatibility, adaptability to canal irregularities, and dimensional stability.^1^ The ideal sealer should promote tissue regeneration and minimize adverse reactions when in contact with periapical tissues.^2^ One endodontic material recognized with impressive biological properties is the mineral trioxide aggregate (MTA), composed of calcium silicates. Over the years, the excellence of MTA for tissue repair has stimulated the development of root canal sealers with calcium silicates as the main component.^3^ The improvement in sealer's composition with dicalcium silicate and tricalcium silicate nanoparticles, more biocompatible radiopacifying agents, such as zirconium oxide, and removal of heavy metals has culminated in the latest generations of endodontic sealers.^4^ According to the excellent biological properties, the peers named this class of material bioceramic.^3^

A bioceramic sealer primarily comprises calcium phosphate, calcium silicate, glass ceramics, aluminum, and zirconia, contributing to superior properties such as biocompatibility and osteogenic potential.^4^, ^5^, ^6^ Currently, various root canal sealers based on calcium silicate are available. One of the newest is AH Plus Bioceramic (AHP-B), which has a composition different from that of other commonly used bioceramics. Unlike its competitors, AHP-B contains fewer silicates than other ready-to-use bioceramics such as Bio-C sealer (BIO-C) and NeoMTA Plus (NEOMTA-P).^7^ Additionally, AH Plus Bioceramic contains only tricalcium silicates and includes dimethyl sulfoxide (DMSO) in its formulation. DMSO, an FDA-approved organosulfur solvent, has been reported to have therapeutic value in osteoarthritis and osteopenia. Studies have shown that DMSO can enhance osteoblastic differentiation of mesenchymal stem cells and prevent bone loss by inhibiting osteoclast activity.^8^

Another curious component of AH Plus Bioceramic is lithium carbonate. Lithium and carbonate groups naturally occur in hydroxyapatite (HA) within mineralized tissue of the human body.^9^ Lithium can reduce the solubility of HA, increase thermal stability compared with other cations, and enhance toughness, osteogenesis, bioactivity, and strength.^10^ However, high concentrations of lithium ions in the serum can be toxic.^11^ Therefore, the effects of lithium and DMSO on this new material's biological properties must be carefully evaluated.^11^ Moreover, AH Plus Bioceramic contains a higher proportion of the zirconia radiopacifier (50 %–70 %), which could interfere with its biological activity when exposed to periapical tissues.^12^

Despite the growing interest in bioceramic sealers, literature comparing the biocompatibility and bioactivity of AHP-B with other bioceramic sealers still needs to be explored. One study demonstrated that AH Plus Bioceramic and Endosequence BC similarly induced gene expression and formation of calcified nodules in human periodontal ligament stem cells.^13^ However, further studies are needed to evaluate its biocompatibility and mineralization potential comprehensively. Therefore, this study aimed to comparatively evaluate the biocompatibility and mineralization potential of the AH Plus® Bioceramic Sealer (AHP-B) with Bio-C® Sealer (Bio-C), NeoMTA Plus® (NEOMTA-P), and MTA-FILLAPEX (MTA-F) in cultures of human dental pulp cells (hDPCs) and osteoblastic cells.

## 2 Methods

### 2.1 Isolation and culture of human dental pulp cells (hDPCs)

The isolation of human dental pulp cells (hDPCs) was approved by the Ethics Committee on Human Research (UnB-FS/CEP no. 23852219.9.0000.0030) and conducted following the ethical principles outlined in the Helsinki Declaration. Pulp samples were collected from the third molars of patients over 18. The tissue was initially placed in a Petri dish containing Dulbecco's Modified Eagle Medium (DMEM) supplemented with 10 % fetal bovine serum (FBS), 100 IU/mL ampicillin, and 100 μg/mL streptomycin for transport to the laboratory (ALL SOLUTIONS GIBCO, GRAND ISLAND, NY). The tissue was washed twice in DPBS 1X, then minced and incubated in trypsin/EDTA (0.25 %) for approximately 5 min maximum. EDTA, a chelating agent, improves the ability of trypsin to detach adherent cells by binding to calcium (Ca^2^⁺) and magnesium (Mg^2^⁺) ions in the extracellular matrix, weakening cell-cell adhesion. Therefore, the cells easily migrate from the tissue fragment to the bottom of the wells as an explant. The culture plates are composed of cell-culture-treated polystyrene, and the surface of the well's bottom is treated with proprietary Nunclon Delta surface; the T-75 flasks have the same treatment, which supports optimal growth and attachment of cells to the growth surface. The cell fragments were transferred to Eppendorf tubes with culture media and centrifuged at 4000 rpm for 3 min. After centrifugation, the pellets of tissue fragments were suspended in fresh media, and the sediment was distributed into 12-well plates with DMEM and incubated at 37 °C, 95 % humidity, and 5 % CO_2__._^14^ Once the cells reached confluence, they were detached using trypsin/EDTA, transferred to T-25 flasks, and sub-cultured at the described conditions. Once confluent, the cells were detached using trypsin/EDTA one more time and maintained in T-75 flasks (ALL PLATES AND FLASKS, CORNING, UNION CITY, CA). For the experiments, the cells were plated at a density of 2 x 10⁴ cells per well in 12-well plates (CORNING, UNION CITY, CA) and incubated under the same conditions described above.

### 2.2 Culture of Saos-2

Human osteoblastic cells (Saos-2, ATCC HTB-85) were cultured as monolayers in T-75 flasks (CORNING, UNION CITY, CA) containing DMEM at 37 °C, 95 % humidity, and 5 % CO_2_. The adhered cells were detached with trypsin/EDTA (0.25 %) at 37 °C for 2 min. The collected cells were placed in 12-well plates (CORNING, UNION CITY, CA) at a density of 2x10^4^/well and incubated under the same conditions described for 24 h before exposure to the materials.^15^

### 2.3 Sealers preparation

The AHP-B, Bio-C, NEOMTA-P, and MTA-F sealers were prepared according to the manufacturers' recommendations. The commercial name, composition, setting time, and percentage of calcium silicate content of each sealer are summarized in Table 1. The sealers were mixed and placed into polypropylene molds (3 × 5mm) for sample preparation. The molds were incubated at 37 °C, 95 % humidity, and 5 % CO_2_ for 72 h to allow the materials to set completely. After the setting period, the hardened sealer samples were carefully transferred into transwell devices containing 0.4 μm permeable membranes (CORNING, UNION CITY, CA). These devices were used to expose the samples to Saos-2 cells (osteoblastic lineage) and human dental pulp cells (hDPCs) in the corresponding experimental setups.

**Table 1:** Comercial name, composition, presentation, manufacturer, setting time, and percentage of silicates.

***Commercial name***	***Composition***	***Presentation***	***Manufacturer***	***Setting time*** ***% of silicates***
**AH Plus®Bioceramic Sealer**	Zirconium dioxide, Tricalcium silicate, Dimethylsulfoxide, Lithium carbonate, Thickening agent	*"Ready to use"*	Dentsply Sirona, Charlotte, USA	120min 5%–15 %^13^
**Bio-C® Sealer**	Tricalcium silicate, dicalcium silicate, tricalcium aluminate, calcium oxide, zirconium oxide, silicon oxide, polyethylene glycol and iron oxide^a^	*"Ready to use"*	Angelus Londrina, PR, Brazil	240min 65 %^a^
**NeoMTAPlus®**	**Dust**: Tricalcium silicate, Dicalcium silicate, tantalum oxide, tricalcium aluminate, calcium sulfate. Liquid: Water-based gel and thickening agents and water-soluble polymers^a^	*powder and gel*	Avalon Biomed Houston, TX, USA	130min 72 %^a^
**MTA-FILLAPEX**	**paste A**: salicylate resin, calcium tungstate, fumed silica. **paste B**: Fumed silica, titanium dioxide, Mineral trioxide aggregate and resin base^a^	*paste-paste*	angelus Londrina, PR, Brazil	100min 13 %^a^

a Manufacturer information.

### 2.4 Calcium ion measurement (Ca^2+^)

After 72 h of setting, the materials samples were immersed in 12-well plates (CORNING, UNION CITY, CA) containing 1.5 mL of DMEM at 37 °C, 95 % humidity, and 5 % CO_2_. The concentration of calcium was determined in parts per million (ppm) with the portable LAQUAtwin Ca-11 calcium meter (HORIBA, SÃO PAULO, SP, BRAZIL). Each measurement and device calibration followed the manufacturer's recommendations. We collected 500 μL of the medium and inserted it into the equipment for measurement at 1, 3, 24, 72, and 96 h. The data were transferred to an Excel spreadsheet (OFFICE 2007, MICROSOFT CORPORATION, REDMOND, WA). The experiment was repeated three times independently (n = 9/group).

### 2.5 MTT assay

After exposure to each material for 24 h, the medium was changed to DEMEM containing 0.55 mg/mL of 3-(4,5-dimethylthiazol-2-yl)-2,5-diphenyltetrazolium (SIGMA CHEMICALS, ST LOUIS, MO, USA) without FBS and the plates were re-incubated for four additional hours. After that time, the formazan crystals were solubilized in 500 μL of acidified isopropanol (HCl: isopropanol, 0.04N), and 100 μL of the sample's solutions were transferred to a 96-well plate (CORNING, UNION CITY, CA). The optical density was measured at 570 nm (ELX800, BIOTEK INSTRUMENTS, WINOOSKI, VT), and the experiment was in triplicates (n = 9/group).

### 2.6 Wound healing assay

Along the bottom of each well of cell culture, about 95 % confluent, we made a 500 μm-wide wound using a sterile P-10 pipette's point. Then, we placed the transwells with sealer samples in the plates for cell exposure. Every 24 h, 30 fields were photographed per group (n = 30/group) using the Zeiss Axiovert 100 inverted microscope (ZEISS, GERMANY, JENA). Thirty fields per group were used in wound healing assays based on statistical sample size calculation to ensure sufficient power to detect meaningful differences with the ANOVA statistical test. The microscopic images were digitized to calculate the percentage of area covered by cells using the ImageJ 1.52K Software (NATIONAL INSTITUTES OF HEALTH, NIH, BETHESDA, MARYLAND, USA).

### 2.7 Alkaline phosphatase assay (ALP)

After five days of exposure, the cells were washed with phosphate buffer (PBS 1X) and immersed in 1 mL of sodium lauryl sulfate (1 mg/mL; SLS, SIGMA CHEMICALS, ST LOUIS, MO) for 30min at room temperature without agitation. Aliquots of each sample (50 μL) were added to the ALP kit components according to the manufacturer's instructions (LABTEST, LAGOA SANTA, MG, BRAZIL). The absorbance was measured at 590 nm (ELx800, Biotek Instruments). We repeated the experiment three times independently (n = 9/group). The ALP was expressed as μmol of thymolphthalein/min/L normalized by the OD of viable cells (570 nm).

### 2.8 Alizarin Red S staining (ARS)

After 15 days of exposure, the cell's monolayers were washed three times with 1X PBS and fixed in 10 % formaldehyde (SIGMA CHEMICALS, ST LOUIS, MO, USA) at room temperature for 15 min. The monolayers were washed twice with distilled water (dH_2_O), and then we added 1 mL of 2 % ARS (pH 4.1) per well (n = 9/group). We incubated the plates at room temperature for 20 min for staining. After that, the wells were washed five times with 2 mL dH_2_O. The stained nodules were microscopically observed and photographed (ZEISS AXIOVERT 100, GERMANY, JENA) with 20x magnification. The calculation of the ARS area followed the ImageJ 1.45 protocol (NATIONAL INSTITUTES OF HEALTH, NIH, USA).

### 2.9 Statistical analysis

The Shapiro-Wilk test was performed to assess the normality of the data, which showed normal distribution for all variables. The data were exported to Excel spreadsheets (OFFICE 2007, MICROSOFT CORPORATION, REDMOND, WA) and subjected to statistical analysis: one-way and two-way analysis of variance (ANOVA), followed by the Bonferroni post-test, with p < 0.05 considered significant. The two-way analysis of variance was used to evaluate the calcium release and the wound healing data according to the levels of “exposure time” and “materials group” as independent variables, and the other results were analyzed using the one-way ANOVA. Sample size calculations were established using the means and within-group variance based on results from other studies^15^^,^^16^ with an alpha of 5 % and 80 % power (“F test for group effect” and “F test for row effect” considering one-way ANOVA and two-way ANOVA, respectively). The studies were performed using STATA/IC 15.1 (STATACORP, COLLEGE STATION, TX, USA).

## 3 Results

### 3.1 Calcium ions release

At 1 h, the MTA-F, Bio-C, and AHP-B groups showed a decrease of calcium in DMEM with values significantly lower than CT (∼97.3 ppm) (Table 2). Oppositely, the NEOMTA-P group had a significant increase of calcium at one (∼160.3 ppm) and 3 h (∼168.33 ppm). The AHP-B bit the calcium concentration in samples only at 72h (∼154.10 ppm), which remained significantly higher than the other groups in 96h of incubation (AHP-B > NEOMTA-P ∼ Bio-C > CT > MTA-F). The Bio-C and NEOMTA-P groups reached similar calcium concentration levels at 72h (∼118 ppm and ∼120 ppm) and were significantly higher than CT. The MTA-F group showed the lowest calcium release throughout the study. Statistics: One-way ANOVA and Bonferroni posthoc, mean ± SD, p < 0.05 (Table 3).

**Table 2:** Comparison of Calcium Concentrations (Ca^2^⁺, ppm) in the Culture Media After Sealer Incubation.

	0h *ppm* ± SD	1 h/ppm ± SD	3h *ppm* ± SD	24 h *ppm* ± SD	72 h *ppm* ± SD	96 h *ppm* ± SD
**CT**	97.8 ± 0,4923^a,A^∗	97.3 ± 0,4923^a,A^∗	99.1 ± 0,4923^a,A^∗	98.7 ± 0,4923^a,A^∗	93.1 ± 0,5149^a,B^∗	88.2 ± 0,7177^a,B^∗
**MTA-F**	97.9 ± 0,4923^a,A^∗	81.6 ± 0,7177^b,B^∗	76.6 ± 0,5222^b,D^∗	29.1 ± 0,7784^b,G^∗	15.9 ± 0,5222^b,J^∗	11.3 ± 0,6030^b,K^∗
**NEOMTA-P**	98.0 ± 0,4082^a,A^∗	160.3 ± 0,5163^c,C^∗	168.3 ± 1,0327^c,C^∗	130.1 ± 0,6324^c,H^∗	120.5 ± 0,8366^c,I^∗	120.9 ± 0,5163^c,I^∗
**BIO-C**	98.1 ± 0,5222^a,A^∗	81.1 ± 0,4922^b,B^∗	110.7 ± 0,^9847d,E^∗	118.2 ± 0,4923^d,I^∗	118.1 ± 0,4923^c,I^∗	119.2 ± 0,9847^c,I^∗
**AHP-B**	97.9 ± 0,5477^a,A^∗	83.6 ± 1,0327^b,B^∗	92.2 ± 1,1690^e,F^∗	97.6 ± 1,2649^a,A^∗	154.1 ± 4,0496^d,C^∗	141.6 ± 1,9407^d,L^∗
			**Two-Way ANOVA**			
***Source of variation***	***SS***	***d.f.***	***MS***	***F***	***p-value***	***F crit***

***Factor #1 (material)***	275.786,18399	5	55.157,23680	4.015,27602	0,00000	2,23684
***Factor #2 (time)***	14.220,41080	5	2.844,08216	207,04037	0,00000	2,23684
***Factor #1 + #2 (material x time)***	195.619,48000	25	7.824,77920	569,61969	0,00000	1,53401
***Within groups***	5.426,05500	395	13,73685			
***Total***	491.052,12979	430	1.141,98170			
***Omega squared for combined effect***	0,98794					

∗Lowercase letters indicate differences between groups within the same time point. Uppercase letters indicate group differences across time points (P < 0.05).

**Table 3:** Comparison of calcium concentrations between groups using the bonferroni post hoc test (P < 0.05).

0h						
Group vs. Group (Contrast)	Difference	95 % Confidence Interval		Test Statistic	p-value	Significance
AHP-B vs BIO-C	−0,04167	−4,50817	4,42484	0,02754	1,00000	Not Significant
AHP-B vs CT	−0,15833	−4,62484	4,30817	0,10464	1,00000	Not Significant
AHP-B vs MTA-F	−0,05833	−4,52484	4,40817	0,03855	1,00000	Not Significant
AHP-B vs NEOMTA-P	−0,37500	−4,94189	4,19189	0,24239	1,00000	Not Significant
BIO-C vs CT	−0,11667	−4,58317	4,34984	0,07710	1,00000	Not Significant
BIO-C vs MTA-F	−0,01667	−4,48317	4,44984	0,01101	1,00000	Not Significant
BIO-C vs NEO	−0,33333	−4,90022	4,23356	0,21546	1,00000	Not Significant
CT vs MTA-F	0,10000	−4,36651	4,56651	0,06609	1,00000	Not Significant
CT vs NEOMTA-P	−0,21667	−4,78356	4,35022	0,14005	1,00000	Not Significant
MTA-F vs NEOMTA-P	−0,31667	−4,88356	4,25022	0,20468	1,00000	Not Significant
**1h**						
*Group vs. Group (Contrast)*	*Difference*	*95 % Confidence Interval*		*Test Statistic*	*p-value*	*Significance*

AHP-B vs BIO-C	2,48333	−1,98317	6,94984	1,64122	1,00000	Not Significant
AHP-B vs CT	−14,31667	−18,78317	−9,85016	9,46180	0,00000	Significant
AHP-B vs MTA-F	1,99167	−2,47484	6,45817	1,31628	1,00000	Not Significant
AHP-B vs NEOMTA-P	−77,19167	−81,65817	−72,72516	51,01554	0,00000	Significant
BIO-C vs CT	−16,80000	−21,26651	−12,33349	11,10303	0,00000	Significant
BIO-C vs MTA-F	−0,49167	−4,95817	3,97484	0,32494	1,00000	Not Significant
BIO-C vs NEO	−79,67500	−84,14151	−75,20849	52,65676	0,00000	Significant
CT vs MTA-F	16,30833	11,84183	20,77484	10,77809	0,00000	Significant
CT vs NEOMTA-P	−62,87500	−67,34151	−58,40849	41,55374	0,00000	Significant
MTA-F vs NEOMTA-P	−79,18333	−83,64984	−74,71683	52,33182	0,00000	Significant
**3h**						
*Group vs. Group (Contrast)*	*Difference*	*95 % Confidence Interval*		*Test Statistic*	*p-value*	*Significance*

AHP-B vs BIO-C	−19,50000	−23,96651	−15,03349	12,88744	0,00000	Significant
AHP-B vs CT	−7,88333	−12,34984	−3,41683	5,21005	4,42488E-6	Significant
AHP-B vs MTA-F	14,60000	10,13349	19,06651	9,64906	0,00000	Significant
AHP-B vs NEOMTA-P	−77,20833	−81,67484	−72,74183	51,02656	0,00000	Significant
BIO-C vs CT	11,61667	7,15016	16,08317	7,67739	1,68937E-12	Significant
BIO-C vs MTA-F	34,10000	29,63349	38,56651	22,53650	0,00000	Significant
BIO-C vs NEO	−57,70833	−62,17484	−53,24183	38,13911	0,00000	Significant
CT vs MTA-F	22,48333	18,01683	26,94984	14,85911	0,00000	Significant
CT vs NEOMTA-P	−69,32500	−73,79151	−64,85849	45,81650	0,00000	Significant
MTA-F vs NEOMTA-P	−91,80833	−96,27484	−87,34183	60,67561	0,00000	Significant
**24h**						
*Group vs. Group (Contrast)*	*Difference*	*95 % Confidence Interval*		*Test Statistic*	*p-value*	*Significance*

AHP-B vs BIO-C	−20,56667	−25,62507	−15,50826	13,59239	0,00000	Significant
AHP-B vs CT	−1,09167	−6,15007	3,96674	0,72148	1,00000	Not Significant
AHP-B vs MTA-F	68,46667	63,40826	73,52507	45,24924	0,00000	Significant
AHP-B vs NEOMTA-P	−32,54167	−37,60007	−27,48326	21,50661	0,00000	Significant
BIO-C vs CT	19,47500	14,41660	24,53340	12,87092	0,00000	Significant
BIO-C vs MTA-F	89,03333	83,97493	94,09174	58,84163	0,00000	Significant
BIO-C vs NEO	−11,97500	−17,03340	−6,91660	7,91421	0,00000	Significant
CT vs MTA-F	69,55833	64,49993	74,61674	45,97071	0,00000	Significant
CT vs NEOMTA-P	−31,45000	−36,50840	−26,39160	20,78513	0,00000	Significant
MTA-F vs NEOMTA-P	−101,00833	−106,06674	−95,94993	66,75584	0,00000	Significant

72h						
*Group vs. Group (Contrast)*	*Difference*	*95 % Confidence Interval*		*Test Statistic*	*p-value*	*Significance*
AHP-B vs BIO-C	35,98333	30,92493	41,04174	23,78118	0,00000	Significant
AHP-B vs CT	61,06667	56,00826	66,12507	40,35862	0,00000	Significant
AHP-B vs MTA-F	138,18333	133,12493	143,24174	91,32459	0,00000	Significant
AHP-B vs NEOMTA-P	33,49167	28,43326	38,55007	22,13446	0,00000	Significant
BIO-C vs CT	25,08333	20,61683	29,54984	16,57743	0,00000	Significant
BIO-C vs MTA-F	102,20000	97,73349	106,66651	67,54341	0,00000	Significant
BIO-C vs NEO	−2,49167	−6,95817	1,97484	1,64673	1,00000	Not Significant
CT vs MTA-F	77,11667	72,65016	81,58317	50,96597	0,00000	Significant
CT vs NEOMTA-P	−27,57500	−32,04151	−23,10849	18,22416	0,00000	Significant
MTA-F vs NEOMTA-P	−104,69167	−109,15817	−100,22516	69,19014	0,00000	Significant
**96h**						
*Group vs. Group (Contrast)*	*Difference*	*95 % Confidence Interval*		*Test Statistic*	*p-value*	*Significance*

AHP-B vs BIO-C	22,43333	17,96683	26,89984	14,82606	0,00000	Significant
AHP-B vs CT	53,41667	48,95016	57,88317	35,30278	0,00000	Significant
AHP-B vs MTA-F	130,25833	125,79183	134,72484	86,08700	0,00000	Significant
AHP-B vs NEOMTA-P	20,69167	16,22516	25,15817	13,67501	0,00000	Significant
BIO-C vs CT	30,98333	26,51683	35,44984	20,47671	0,00000	Significant
BIO-C vs MTA-F	107,82500	103,35849	112,29151	71,26094	0,00000	Significant
BIO-C vs NEO	−1,74167	−6,20817	2,72484	1,15106	1,00000	Not Significant
CT vs MTA-F	76,84167	72,37516	81,30817	50,78423	0,00000	Significant
CT vs NEOMTA-P	−32,72500	−37,19151	−28,25849	21,62777	0,00000	Significant
MTA-F vs NEOMTA-P	−109,56667	−114,03317	−105,10016	72,41200	0,00000	Significant

### 3.2 Cell viability

**Fig. 1:**
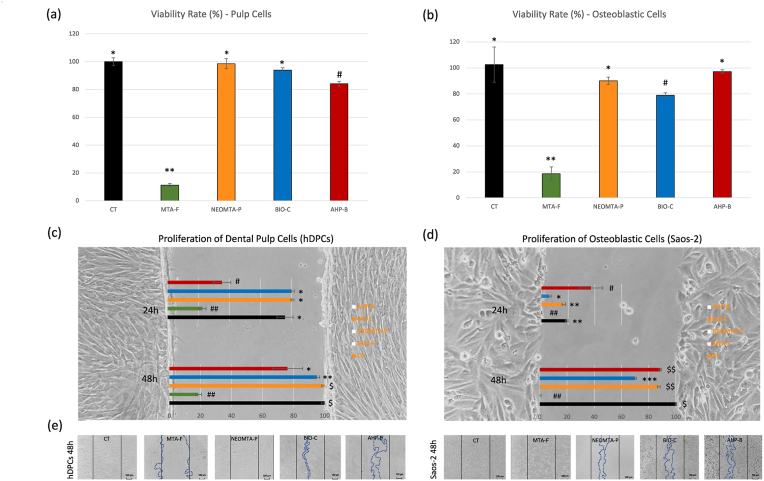
Cellular viability and proliferation towards the wound of hDPCs and Saos-2 exposed to NEOMTA-P, BIO-C, AHP-B, and MTA-F. **Statistics:** one-way and two-way ANOVA, followed by Bonferroni post-hoc tests. The material type and exposure time were significant factors in the two-way ANOVA of the wound healing assay. Statistical differences between groups are indicated by different symbols (mean ± SD, p < 0.05).

After 24 h of exposure to the AHP-B sealer, the viability of human dental pulp cells (hDPCs) was significantly lower (∼84.11 %) compared to the NEOMTA-P, Bio-C, and control (CT) groups, which showed viability rates of approximately ∼98.51 %, ∼93.92 %, and ∼100.01 %, respectively (Fig. 1a). In contrast, osteoblastic Saos-2 cells exposed to AHP-B (∼97 %) and NEOMTA-P (∼90 %) demonstrated viability rates comparable to the control group (∼102 %) (Fig. 1b). The viability of Saos-2 cells exposed to Bio-C (∼78 %) was significantly lower than that of the CT group. Additionally, both hDPCs and Saos-2 cells exposed to MTA-F exhibited the lowest viability rates among all groups.

### 3.3 Cell proliferation

After 24 h of exposure, the AHP-B group showed approximately 35 % wound coverage by hDPCs, significantly lower than the NEOMTA-P, Bio-C, and control (CT) groups, which exhibited near 80 % coverage (Fig. 1c). At 48 h, the statistical difference between AHP-B and the other groups persisted: the CT and NEOMTA-P groups achieved nearly 100 % wound closure, Bio-C covered about 95 %, while AHP-B reached only ∼77 %. At 48 h, only NEOMTA-P and CT entirely covered the wound area, whereas AHP-B remained at a lower level of coverage.

In contrast, the percentage of wound area covered by Saos-2 cells after 24 h of exposure to AHP-B was significantly higher (∼37.09 %) compared to NEOMTA-P (∼16.62 %) and Bio-C (∼5.82 %) (Fig. 1d). By 48 h, both AHP-B (∼88.7 %) and NEOMTA-P (∼87.5 %) groups showed similar cell coverage, with the CT group achieving ∼100 % wound closure. The Bio-C group, however, exhibited significantly lower coverage (∼70 %). Notably, except for the MTA-F group, hDPCs and Saos-2 cells in the AHP-B, NEOMTA-P, and Bio-C groups remained confluent and exhibited normal morphology in the area surrounding the wound (Fig. 1e). Despite the differences in proliferation rates, cells from these groups were attached and displayed typical morphology (100 μm scale bars) (Fig. 1e). Conversely, most cells exposed to MTA-F detached and exhibited abnormal shapes. Cell viability was analyzed using one-way ANOVA with Bonferroni post-hoc tests (P < 0.05).

### 3.4 Alkaline phosphatase activity

**Fig. 2:**
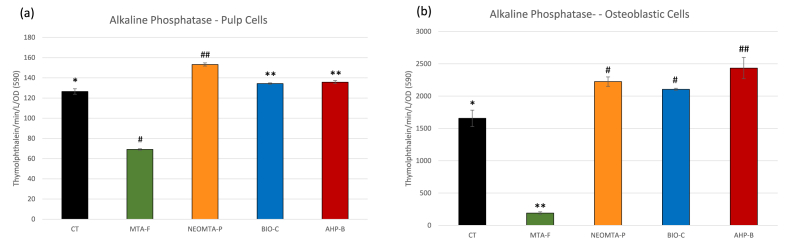
Alkaline phosphatase activity (ALP) of human dental pulp cells (hDPCs) and osteoblastic cells (Saos-2) exposed to AHP-B, NEOMTA-P, BIO-C, and MTA-F. **Statistics:** One-way ANOVA followed by Bonferroni post-hoc tests. Different symbols represent statistically significant group differences (mean ± SD, p < 0.05).

Alkaline phosphatase (ALP) activity in hDPCs exposed to NEOMTA-P (∼153.2 thymolphthalein/min/L/OD) was the highest, followed by AHP-B (∼135.7 thymolphthalein/min/L/OD) and Bio-C (∼134.2 thymolphthalein/min/L/OD), which were statistically similar (Fig. 2a). All hDPC groups exhibited significantly higher ALP activity compared to the control (CT) group (∼126.4 thymolphthalein/min/L/OD).

In osteoblastic cells, ALP activity was also significantly higher in the AHP-B (∼2434.2 thymolphthalein/min/L/OD), NEOMTA-P (∼2224.3 thymolphthalein/min/L/OD), and Bio-C (∼2104.5 thymolphthalein/min/L/OD) groups compared to CT (∼1656.6 thymolphthalein/min/L/OD) (Fig. 2b). The MTA-F group exhibited the lowest ALP activity in both hDPCs and Saos-2 cells. After five days of exposure to the sealers, ALP activity in both hDPCs and Saos-2 cells was significantly higher than in the CT group, except for MTA-F. The hDPCs exposed to NEOMTA-P showed the highest ALP activity (Fig. 2a), while AHP-B induced the highest ALP activity in Saos-2 cells (Fig. 2b).

### 3.5 Mineralization

**Fig. 3:**
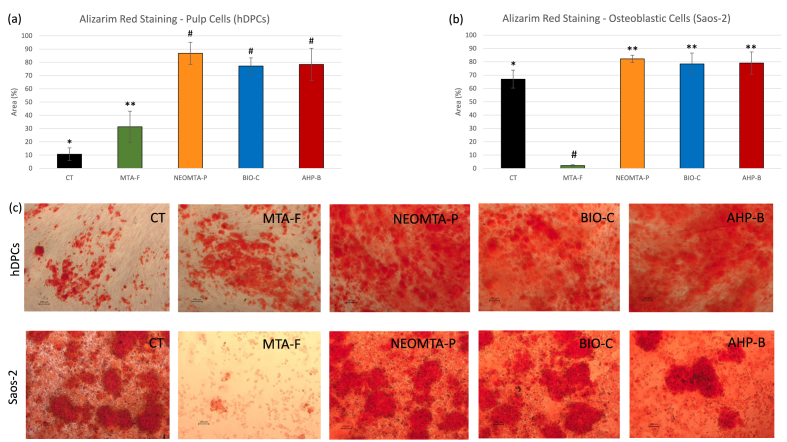
Mineralization in cultures of hDPCs and Saos-2 as a late marker of osteoblastic differentiation. **Statistics:** Data were analyzed using one-way ANOVA followed by Bonferroni post-hoc tests. Different symbols indicate statistically significant group differences (mean ± SD, p < 0.05).

hDPCs exposed to AHP-B (∼78.41 %), NEOMTA-P (∼86.79 %), and Bio-C (∼77.22 %) in an osteogenic medium for 15 days demonstrated robust Alizarin Red S (ARS) staining and a significantly higher percentage of mineralized areas compared to the control (CT) group (∼10.7 %) (Fig. 3a and c). The MTA-F group exhibited a significantly lower mineralized area (∼31.3 %) than the other groups, with values similar to the CT group (∼10.7 %). A similar pattern of ARS staining was observed in Saos-2 cells, with NEOMTA-P (∼82.23 %), AHP-B (∼79.05 %), and Bio-C (∼78.39 %) showing significantly greater mineralized areas compared to CT (∼67 %) (Fig. 3b). In contrast, the MTA-F group displayed the lowest mineralized area (∼2.21 %), along with fewer surviving cells (Fig. 3c).

## 4 Discussion

In this study, the bioceramic sealers differently affected hDPCs and Saos-2. When placed within the root canal, the materials employed in endodontic treatment should strive to maintain minimal contact with periradicular tissues. Nevertheless, throughout the treatment, there is a possibility of these sealers extravasating through the apical foramina, lateral canals, or accessory canals, which may still contain vital pulp cells, potentially impacting the tissues to varying extents.^12^ Hence, the sealer must not induce harm to these tissues or hinder the tissue healing process.^17^ The sealer's composition is likely responsible for the cellular behavior, especially AHP-B, which has lithium carbonate, dimethyl sulfoxide (DMSO), and less tricalcium silicate. Lithium is an osteoprotective agent that activates signaling pathways, such as the BMP-2 (bone morphogenetic protein-2), Wnt/β-catenin (Wingless/beta-catenin), and PI3K/Akt (phosphatidylinositol 3-kinase/protein kinase B).^18^ DMSO enhances the absorption of other compounds across cell membranes, increasing their effectiveness^19^ and the bioavailability of calcium.^20^

Interestingly, MTA-F, AHP-B, and BIO-C groups showed an immediate decrease in media calcium (Ca^2+^) at 1h of incubation. The balance between the sealer hydration and its concentration of calcium silicates (CaSi) may explain this result.^21^ During the early stages of hydration, the sealer forms a thin layer of byproducts on its surface, which adsorb or react with Ca^2+^ from the media, reducing Ca^2+^ in the supernatant.^22^ Different from the other sealers, NEOMTA-P has the highest content of CaSi (NEOMTA-P, 72 % > BIO-C, 65 % > AHP-B, 15 % > MTA-F, 13 %; Table 1) and leached a significant amount of Ca^2+^ already at the first hours of immersion in the culture media. NEOMTA-P showed a solubility of ∼9 % and a Ca^+2^ release of 126 ppm after 24 h of incubation in deionized water.^23^^,^^24^ In our study, BIO-C released an increased concentration of Ca^2+^ at 3 h of incubation and sustained a release of over 110 ppm until 96 h in DMEM. In a different study, BIO-C solubility was ∼11.4 %,^25^ and this sealer released 157.3 ppm of Ca^+2^ after 24 h in DMEM,^24^ corroborating our results. In the AHP-B group, the Ca^2+^ concentration started to increase at 3h, the Ca^2+^, reaching a high concentration at 72h, which may correlate with the sealer solubility during the prolonged setting time and Ca^2+^ release to the media from 24 to 72 h.^21^^,^^22^ In a study of physical-chemical analysis,^22^ the AHP-B release of calcium was similar to our research (∼30.6 ppm at 24 h of immersion in distilled water and a peak of ∼67.5 ppm at 72 h). The authors attributed this result to the low percentages of tricalcium silicate and the overall AHP-B composition. According to them, “the ion release depends on the nature of the network structure of the sealer responsible for water absorption and solubility as well as the permeability of the material to water diffusion”.^22^ The sealer composition may lead to a high volume of open pores; consequently, this material can absorb more water, show higher solubility, and potentially release higher ions depending on the content of reactive CaSi particles.^22^ In that study, AHP-B, the sealer with the lowest proportion of calcium silicate, showed higher solubility, open pore volume, and water absorption than AH Plus, Ceraseal, and NeoSealer Flo. MTA-F showed a progressive decrease of Ca^2+^ in the media until 96h. Possibly, MTA-F adsorbed Ca^2+^ from the culture media during the entire experiment. The solubility of MTA-F is still controversial.^26^ The decrease of Ca^2+^ at 96h that NEOMTA-P, BIO-C, and AHP-B showed possibly correlates with the sealer complete set.

After 24 h of exposure, all sealers proved to be biocompatible with both hDPCs and Saos-2, with the exception of MTA-F. The hDPCs and Saos-2 cells exhibited approximately 100 % viability after being exposed to NEOMTA-P, which had previously demonstrated an absence of cytotoxicity in another study involving dental pulp stem cells.^27^ However, the viability rate of hDPCs exposed to AHP-B was significantly lower at around 80 %, while Saos-2 cells maintained approximately 100 % viability. Conversely, exposure to BIO-C resulted in hDPCs displaying around 100 % viability, whereas Saos-2 cells showed a viability rate of approximately 78 %. The longer setting times noted for both AHP-B and BIO-C may contribute to increased solubility and a higher release of sealer compounds, particularly resinous compounds, which could lead to cytotoxicity and adversely affect cell viability.^28^ Both AHP-B and BIO-C exhibited mild initial cytotoxicity towards human periodontal ligament fibroblasts.^28^ Despite the lower viability rates of hDPCs exposed to AHP-B and Saos-2 cells exposed to BIO-C, most cells retained normal morphology in the wound assay. According to ISO 10993-5, neither BIO-C nor AHP-B was cytotoxic. A viability rate lower than 70 % or more than 20 % of cells showing morphological alterations would indicate cytotoxicity, as observed in the MTA-F group.^28^

The growth of hDPC's was not significantly affected by NEOMTA-P or BIO-C in the wound assay. However, the hDPCs exposed to AHP-B exhibited a higher percentage of uncovered areas, supporting the MTT assay findings. In contrast, Saos-2 in the AHP-B group showed the highest coverage at 24h, suggesting cell differentiation: an initial phase of cell proliferation followed by a halt in proliferation and the synthesis of extracellular matrix.^16^ Saos-2 treated with BIO-C and NEOMTA-P showed a slowdown in proliferation towards the wound area at 24 and 48 h, suggesting that osteoblastic cells may begin differentiation earlier when exposed to BIO-C and NEOMTA-P. It is crucial to note that BIO-C and NEOMTA-P released high amounts of calcium ions early (1 and 3 h), while AHP-B released calcium ions from 24 to 72 h. Calcium silicate interacts with environmental fluids, releasing calcium ions (Ca^2^⁺) into the medium. The amount of calcium released might be higher due to a more significant proportion of calcium silicate or the specific additives used in the formulations.^24^ Calcium accelerates tissue healing by promoting restorative dentinogenesis, recruiting and activating hard tissue-producing cells.^29^ The increased calcium signaling from ion release into the media may explain the observed halt in the proliferation of osteoblastic cells in favor of differentiation at the first hours of sealer incubation.

Moreover, the DMSO from AHP-B could trigger the release of Ca^+2^ from intracellular stores,^20^ interfering with the mitochondrial dehydrogenase activity (MTT) and Wound results. Lithium, another component of AHP-B, can activate canonical Wingless (Wnt)/beta (b)-catenin, phosphatidylinositol 3-kinase (PI3K)/protein kinase B (Akt), and bone morphogenetic protein-2 (BMP-2) transduction pathways, promoting osteoblastic activities and increasing osteoblasts proliferation.^18^ Lithium, DMSO, and Ca^2+^ properties may explain the osteoblastic cells' response to AHP-B in this study. However, a study showed that the lithium effect is concentration-dependent on human periodontal ligament cells (hPDLCs).^30^ Ideal concentrations of lithium-calcium-silicate activated the gene expression of mineralization markers in hPDLCs. However, high concentrations slowed down proliferation and significantly impacted the ALP activity.^30^ Possibly, the proportion of lithium-calcium-silicate that leached from AHP-B affected the hDPCs similarly, slowing down proliferation.

In osteoblastic cells exposed to AHP-B, the ALP was higher than NEOMTA-P and BIO-C, while the ALP activity in hDPCs was significantly higher in the NEOMTA-P group. ALP activity is a very early marker of osteoblastic differentiation, and the sealers' Ca^2+^ release after 72h, the DMSO, and lithium of AHP-B may also explain such results. Human osteoblasts cultured in osteogenic media supplemented with increasing concentrations of DMSO had enhanced ALP and mineralization.^31^ During mineralization, the building Ca^2+^ gradient first activates ALP, but very high Ca^2+^ concentrations gradually inactivate the enzyme, as Ca^2+^ competes with zinc for ALP M1 and M2 catalytic domains.^32^ A study that evaluated the crystal structure and biological responses of dental pulp stem cells (hDPSCs) to bone grafts doped with lithium showed that lithium ions favored the entrance of the β-tricalcium phosphate at the calcium sites and calcium vacancy sites.^12^ However, lithium concentrations higher than 10 % induced crystal instability, burst the release of lithium ions and showed no improvement in the ALP or mineralization potential of hDPSCs.^12^ The increase in alkaline phosphatase (ALP) enzyme expression in cells exposed to calcium silicate-based cement while concurrently maintaining cellular proliferation suggests the potential capability of these materials to promote osteogenic differentiation.^27^ Prolonged treatment with lithium chloride (LiCl) in the culture of mice dental pulp cells inhibited differentiation.^31^ The LiCl inhibition on hDPCs osteogenesis was not mediated through the Wnt/β-catenin pathway, suggesting that reparative dentinogenesis may involve multiple pathways.^32^ Such results may explain the behavior of hDPCs exposed to AHP-B in our study. In summary, the novel calcium silicate-based bioceramic sealer, AHP-B, did not stimulate the hDPCs during early differentiation as the NEOMTA-P and BIO-C sealers did, although it stimulated the osteoblastic cells.

Based on scientific literature, this in vitro study tries to simulate the clinical environment. However, one limitation of this study is that certain variables may, in vivo, alter the performance of the evaluated sealers, and it does not fully reflect the complexity of in vivo conditions. The absence of systemic factors and the short duration of cell exposure limit the extrapolation of the results to long-term clinical scenarios. Moreover, one may consider the diversity of cells that will respond to the endodontic sealers in vivo, osteoblasts and remaining dental pulp cells, periodontal ligament cells, osteoclasts, and macrophages, among others. Indeed, there are some uncertainties regarding the behavior of bioceramic materials in vivo, particularly in the long term. Ultimately, in vitro and in vivo research are complementary and essential for advancing scientific knowledge and developing new therapeutic approaches.

## 5 Conclusion

In this study, AHP-B, NEOMTA-P, and BIO-C stimulated osteogenesis in hDPCs and Saos-2 with notable differences. Considering the cell proliferation and the ALP activity, AHP-B showed an improved early stimulation of osteoblastic cells, while hDPCs were more responsive to NEOMTA-P. All the sealers, except MTA-F, leached high amounts of Ca^2+^ to the media over varying time points. Calcium triggers crucial pathways for osteoblast differentiation. This property is relevant when choosing an endodontic sealer for root canal obturation. For instance, in cases of pulp vitality, like irreversible pulpitis, aiming the fast reeling of the adjacent tissue in the apical region and the remaining pulp tissue in the inaccessible root canal system, an endodontic sealer that releases high amounts of calcium at the first hours and promotes mineralization might be preferable. Conversely, materials that later and continuously leach Ca^2+^ could be more suitable in scenarios involving bone defects and apical lesions, permitting the control of adjacent tissue inflammation and longer stimulating mineralization. This hypothesis deserves further evaluation in a clinical trial.

## Funding and support

This work was supported by the University of Brasília (PROAP/DGP), Brazil.

## Declaration of competing interest

The authors declare that they have no known competing financial interests or personal relationships that could have appeared to influence the work reported in this paper.

## References

[1] Zhou H., Shen Y., Zheng W., Li L., Haapasalo M. (2021). A comprehensive review of endodontic sealers: properties, components, and clinical applications. J Endod.

[2] Nair P.N.R., Love R.M. (2021). Biocompatibility and tissue regeneration in endodontic sealers. J Endod.

[3] Torabinejad M., Pitt Ford T.R., McKendry D.J., Abedi H.R., Miller D.A., Kariyawasam S.P. (1997). Histologic assessment of mineral trioxide aggregate as a root-end filling in monkeys. J Endod.

[4] Alves Silva E.C., Tanomaru-Filho M., da Silva G.F., Delfino M.M., Cerri P.S., Guerreiro-Tanomaru J.M. (2020). Biocompatibility and bioactive potential of new calcium silicate-based endodontic sealers: Bio-C sealer and sealer plus BC. J Endod.

[5] Koch K. (2011). Bioceramic technology a game changer in endodontic obturation. Art of Dentistry.

[6] Koch K.A., Brave D.G. (2012). Bioceramics, Part II: the clinician's viewpoint. Dent Today.

[7] Donnermeyer D., Schemkämper P., Bürklein S., Schäfer E. (2022). Short and long-term solubility, alkalizing effect, and thermal persistence of premixed calcium silicate-based sealers: AH plus bioceramic sealer vs. Total fill BC sealer. Materials.

[8] Yang C., Madhu V., Thomas C. (2015). Inhibition of differentiation and function of osteoclasts by dimethyl sulfoxide (DMSO). Cell Tissue Res.

[9] Hajji H., Le Gallet S., Saviot L., Ben Salem E., Millot N. (2022). Mechanosynthesis of carbonate and lithium co-substituted hydroxyfluorapatite. Mater Res Bull.

[10] Matsumoto N., Yoshida K., Hashimoto K., Toda Y. (2009). Thermal stability of β-tricalcium phosphate doped with monovalent metal ions. Mater Res Bull.

[11] Yoo K.H., Kim Y., Kim Y.I., Bae M.K., Yoon S.Y. (2022). Lithium doped biphasic calcium phosphate: structural analysis and osteo/odontogenic potential in vitro. Front Bioeng Biotechnol.

[12] Queiroz M.B., Torres F.F.E., Rodrigues E.M. (2021). Physicochemical, biological, and antibacterial evaluation of tricalcium silicate-based reparative cements with different radiopacifiers. Dent Mater.

[13] Sanz J.L., López‐García S., Rodríguez‐Lozano F.J. (2022). Cytocompatibility and bioactive potential of AH Plus Bioceramic Sealer: an in vitro study. Int Endod J.

[14] Patil R., Kale A.D., Mane D.R., Patil D. (2020). Isolation, culture and characterization of primary cell lines of human buccal mucosal fibroblasts: a combination of explant enzamytic technique. J Oral Maxillofac Pathol.

[15] Santiago M.C., Gomes-Cornélio A.L., de Oliveira L.A., Tanomaru-Filho M., Salles L.P. (2021). Calcium silicate-based cements cause environmental stiffness and show diverse potential to induce osteogenesis in human osteoblastic cells. Sci Rep.

[16] Gomes-Cornélio A.L., Rodrigues E.M., Salles L.P. (2015). Bioactivity of MTA plus, Biodentine and an experimental calcium silicate-based cement on human osteoblast-like cells. Int Endod J.

[17] Aminoshariae A., Kulild J.C. (2020). The impact of sealer extrusion on endodontic outcome: a systematic review with meta-analysis. Aust Endod J.

[18] Wong S.K., Chin K.Y., Ima-Nirwana S. (2020). The skeletal-protecting action and mechanisms of action for mood-stabilizing drug lithium chloride: Current evidence and future potential research areas. Front Pharmacol.

[19] Hoang C., Nguyen A.K., Nguyen T.Q. (2021). Application of dimethyl sulfoxide as a therapeutic agent and drug vehicle for eye diseases. J Ocul Pharmacol Ther.

[20] Koutroulis A., Kuehne S.A., Cooper P.R., Camilleri J. (2019). The role of calcium ion release on biocompatibility and antimicrobial properties of hydraulic cements. Sci Rep.

[21] Morley P., Whitfield J.F. (1993). The differentiation inducer, dimethyl sulfoxide, transiently increases the intracellular calcium ion concentration in various cell types. J Cell Physiol.

[22] Zamparini F., Prati C., Taddei P., Spinelli A., Di Foggia M., Gandolfi M.G. (2022). Chemical-physical properties and bioactivity of new premixed calcium silicate-bioceramic root canal sealers. Int J Mol Sci.

[23] Siboni F., Taddei P., Prati C., Gandolfi M.G. (2017). Properties of NeoMTA plus and MTA plus cements for endodontics. Int Endod J.

[24] Sanz J.L., López-García S., Lozano A. (2021). Microstructural composition, ion release, and bioactive potential of new premixed calcium silicate-based endodontic sealers indicated for warm vertical compaction technique. Clin Oral Investig.

[25] Silva E.M., Alcalde M.P., Vivan R.R., Pomini M., Tanomaru-Filho M., Duarte M.A.H. (2022). Evaluation of in vitro experimental model for analysis of bioceramic sealers. Braz Oral Res.

[26] Amoroso-Silva P.A., Guimarães B.M., Marciano M.A. (2014). Microscopic analysis of the quality of obturation and physical properties of MTA Fillapex. Microsc Res Tech.

[27] Sismanoglu S., Ercal P. (2023 Apr). Effects of calcium silicate-based cements on odonto/osteogenic differentiation potential in mesenchymal stem cells. Aust Endod J.

[28] Kwak S.W., Koo J., Song M., Jang I.H., Gambarini G., Kim H.C. (2023). Physicochemical properties and biocompatibility of various bioceramic root canal sealers: in vitro study. J Endod.

[29] Khoswanto C., Dewi I.K. (2024). MTA as modulator of periapical tissue healing in rat molar: a histological study. J Oral Biol Craniofac Res..

[30] Zhang QChen L., Chen B., Chen C. (2019). Lithium-calcium-silicate bioceramics stimulating cementogenic/osteogenic differentiation of periodontal ligament cells and periodontal regeneration. Appl Mater Today.

[31] Stephens A.S., Stephens S.R., Hobbs C. (2011). Myocyte enhancer factor 2c, an osteoblast transcription factor identified by dimethyl sulfoxide (DMSO)-enhanced mineralization. J Biol Chem.

[32] Hoylaerts M.F., Van Kerckhoven S., Kiffer-Moreira T., Sheen C., Narisawa S., Millán J.L. (2015). Functional significance of calcium binding to tissue-nonspecific alkaline phosphatase. PLoS One.

